# Laryngology

**Published:** 1903-12

**Authors:** P. Watson Williams


### LARYNGOLOGY.

A recent discussion[1] on "**the upper respiratory tract as a source of systemic infection**" touches on many points of great interest to the general medical practitioner. In introducing this topic at the Annual Meeting of the British Medical Association at Swansea, de Havilland Hall referred firstly to the cases of typhoid fever which arose from infection through the respiratory tract, mentioning particularly a case which occurred in Bristol, reported in 1894 by the writer of this review. Hence in cases of typhoid fever with cough and expectoration, the latter should be regarded as infective, and be disinfected accordingly.

After reviewing the evidence in favour of influenza and whooping cough being contracted by infection of the nose or throat, Hall directed attention to the nasal fossæ as the frequent seat of primary diphtheria, often of a latent type, and forming the source of infection for others. Too often patients who have had slight operative treatment of the nasal passages, *e.g.* cauterisation of the mucosa, are allowed to go about their usual routine work without any warning; and it is significant that Hall has had two instances in his own experience where, after the application of the galvano-cautery, scarlatina was contracted, and as he believes, the germ entered the system through the wound produced.

The part played by the faucial tonsils in rheumatic fever and in acute endocarditis merits wider appreciation. De Havilland Hall stated that "the intimate connection which exists between the throat and acute rheumatism is shown by the frequency with which tonsillitis precedes or accompanies an attack of acute rheumatism. I think that there is a great deal to be said in favour of the view that tonsillitis is a primary infective disease of the lacunæ, and that rheumatic fever is a secondary disease arising from the absorption of microbes or their products into the system." He referred further to instances of pericarditis apparently due to infection arising through the nose and the faucial tonsils. Jobson Horne cited cases of malignant endocarditis in which the primary infection was in the larynx, the facts

[1] *Brit. M. J.*, 1903, ii. 1137.

being revealed or confirmed by the necropsies. He showed that the usual starting-point of the laryngeal ulceration was in a fold of mucous membrane, commencing behind the vocal process and passing obliquely upwards and backwards, to be lost between the summits of the cartilages of Santorini and Wrisberg. He has found this to be a site which lends itself to infection in other diseases; here secretion may tend to stagnate, and here stress and strain is put upon the laryngeal mucosa, and a breach in the inflamed surface would readily occur. This point he terms the "vulnerable spot." Horne's observations in a large number of cases dying from phthisis, including an elaborate and painstaking investigation of the larynx, led him to the conclusion that—

1. When the larynx is infected with tubercle the disease is already established in the lung.

2. That by the time the disease in the larynx has advanced to ulceration the disease in the lung has advanced to cavitation.

3. When the disease in the lung is confined to the pure miliary form the larynx is never infected.

He is firmly convinced that the infection of the larynx takes place by the sputum. It is unnecessary to do more than allude to the remarks of de Havilland Hall reiterating the evidence pointing conclusively to the naso-pharyngeal lymphoid ring as a path of invasion of tubercle, both to the cervical glands and the lungs. In the course of the discussion, among much interesting matter brought forward by Goodall, we note that in his experience tuberculous cervical lymphadenitis is in a large number of cases associated with dilatation of the lacunæ, which on close inspection show a narrow hyperæmic halo about their orifice; the tonsils, at the same time, are not necessarily enlarged. Where removal of such tonsils cannot be practised, he advises reduction of the size of the lacunæ by cauterisation or by the application of iodine.

The President expressed his decided opinion that infective processes often remained localised in the tonsils and other lymphoid tissues in or connected with the throat, and that these lymphoid aggregations, often a source of danger, were when healthy a valuable protective barrier which should not be heedlessly removed without good reason.

One more point of practical importance bearing on the question of rheumatic infections through the upper respiratory tract was adduced by Poynton, whose investigations had shown that the diplococci in the tonsils in rheumatic tonsillitis were identical with those isolated from the blood in rheumatic fever. But in many cases when once the micro-organisms have gained access to the general system they make themselves new homes in the tissues, hence every effort should be made to arrest the process in the upper respiratory tract before it generalises.

P. Watson Williams.

